# Supplementary material for: Are carbon emissions trading and green financial instruments synergistic? -Comprehensive quantitative research based on content analysis
Source: PLoS One. 2024 Mar 7;19(3):e0298601. doi: 10.1371/journal.pone.0298601 (PMC10919601; doi:10.1371/journal.pone.0298601)
Supplement: S1 Data — (ZIP) [file pone.0298601.s001.zip › Data/地级市Z_F工作报告.zip!/║■─╧/╗│╗»╩╨/└┤╫╘╣1⁄2╓┌║┼í╛┬φ┐╦╩2╛▌═°í┐_2019.docx]

各位代表：

现在，我代表市人民政府向大会报告工作，请予审查，并请各位政协委员和其他列席人员提出意见。

一、2018年工作回顾

过去的一年，我们坚持以习近平新时代中国特色社会主义思想为指导，认真贯彻党的十九大和十九届二中、三中全会精神，在省委省政府和市委的坚强领导下，对标对表，攻坚克难，有效应对各种困难和挑战，较好完成了全年目标任务，呈现出稳中有进、进中向好的高质量发展态势。实现地区生产总值增长8%（预计数，下同），固定资产投资增长12%，规模工业增加值增长7.3%，一般公共预算收入增长10%，社会消费品零售总额增长10%，城乡居民人均可支配收入分别增长8.5%、10.8%。

（一）三大攻坚战取得新成效。始终把打好三大攻坚战作为推动高质量发展的首要任务，用心用脑、精准精细，集中精力纵深推进。防范化解重大风险整体可控。厘清政府债务底数，制定政府债务化解方案，减少隐性债务19.31亿元。“停缓调撤”项目329个，压缩投资543.04亿元。开展“三资”清理，清理市本级土地2049亩、政府资产3万平米、政府借贷资金76.69亿元。闲置土地处置率达91.22%。实现城乡建设用地增减挂钩指标收益23.09亿元。推进平台公司转型，融资平台由87家减少至30家。新增信贷投放89.99亿元，贷存比56.23%。银行不良贷款率下降至3.62%。精准脱贫成效明显。圆满完成年度目标任务，实现16.33万贫困人口稳定脱贫，419个贫困村出列，贫困发生率下降至2.32%，辰溪、芷江、新晃、会同、靖州5个计划脱贫摘帽县通过省级实地检查。完成易地扶贫搬迁5864户23456人，提前两年完成“十三五”任务。发放贫困学生资助资金1.28亿元，惠及31.57万人次。贫困人口住院实际报销比例达85%以上。完成贫困村农网改造411个。新建改建“四好农村路”5645公里，芷江获评“四好农村路”全国示范县。扎实开展脱贫攻坚作风建设年活动和扶贫领域腐败问题专项治理。污染防治强力推进。全面打响蓝天碧水净土保卫战，集中开展“夏季攻势”行动，城区空气质量优良率达92.1%。整治黑臭水体9条，清理“僵尸船”326艘；城东污水处理厂顺利开工建设，中方、辰溪、洪江市工业集中区污水处理设施投入运营；37个地表水考核断面水质达到考核要求，14个集中式饮用水源地水质达标率100%；完成重金属污染防治项目16个，整治尾矿库及冶炼企业28家，矿山恢复治理87.19公顷。中央环保督察“回头看”、省环保督察组交办件办结率100%。

（二）产业发展实现新提升。按照省委省政府产业项目建设年活动要求，全面启动产业及产业园区三年提升行动，实施重点产业项目215个，投资347亿元。三次产业比由13.9：35.8：50.3调整为12.0：34.0：54.0。工业经济得到优化。新增规模工业企业110家，超省定任务55家。园区规模工业增加值占规模工业比重达60%。辰州矿业、千源铝业、怀化远大、五新钢模等企业产值增长均超20%。新兴产业快速发展。亚信电子、奇力新电子等企业日益壮大。全市新材料企业达24家，实现主营业务收入43亿元。中药材种植面积44万亩，综合产值80亿元。规模以上文体企业达155家。第三产业蓬勃发展。成功入选国家第三批“多式联运示范工程”，传化智能公路物流港、佳惠·武陵山农产品（冷链）物流产业园等30个重点项目进展顺利，新增限额以上商贸流通企业159家、重点商贸服务企业25家。积极创建国家全域旅游示范市，通道芋头古侗寨获评4A级旅游景区，黄岩旅游区、九丰农博园和穿岩山景区通过4A级旅游景区景观质量评定，洪江古商城创建5A级旅游景区步伐加快；宝中旅游国际旅行社获评省五星级旅行社，实现零的突破；具备五星级条件的酒店顺利开工；全年接待游客5800万人次，实现旅游收入450亿元，分别增长16%、15%。成功获批全国电子商务进农村综合示范整体推进市，实现电子商务交易额180亿元，增长35%。成功举办汽车展销节、生态文化旅游节、健康产业和文体产业博览会等节会活动，消费对经济的贡献率达59.5%。

（三）改革开放展现新气象。始终把创新引领开放崛起作为推动高质量发展的关键举措，向改革要动力，向开放要活力。供给侧结构性改革纵深推进。9大类200余项重点改革任务全面完成。“三去一降一补”取得明显成效，全年关闭非煤矿山29家，商品房去库存525万平米，为企业减税降费4.8亿元。集体林业综合改革经验在全国推介，成功入选新一轮国家级试验区。商事制度改革全面落实，新登记市场主体3.37万户，数量实现三年翻番。市本级不动产统一登记改革进展顺利。农村殡葬制度改革加快推进。城市公立医院综合改革效果评价考核通过国家复评。全方位开放格局加快构建。深入开展“五大开放行动”，成功引进500强企业10家，招商引资省外境内资金270亿元，实际利用外资6000万美元，实现进出口总额8500万美元。注重开放载体和平台建设，怀化海关获批成立，铁路口岸建成开关，舞水公共保税仓、跨境商品展示交易中心正式运营，成功入列商贸服务型国家物流枢纽承载城市。怀化商标受理中心辐射带动效应增强，注册商标总量1.2万余件，创建中国驰名商标3件。经开区外贸产业孵化园成功开园。怀化至明斯克中欧班列正式开通。怀邵衡铁路、怀芷高速建成通车，张吉怀客运专线、渝怀铁路增建二线加快推进。芷江机场旅客年吞吐量突破50万人次。创新体系和创新能力建设持续加强。认真落实创新是第一动力的要求，扎实推动生态创新融合发展。怀化高新区成功获批国家级高新区，怀化国家农业科技园区通过国家验收，洪江（洪江市、洪江区）工业集中区获批省级高新区。拥有各类科技创新创业平台载体近400个，其中省级以上60个。高新技术企业增至159家、增长115%，增速居全省前列；实现高新技术产业增加值210亿元。R&D经费支出达24亿元，增长94.9%。发明专利年申请量达1364件，增长128.5%，增速全省第一。认真落实人才是第一资源的要求，启动五溪人才行动计划，全市引进高层次、紧缺人才860人。侯卫东官场笔记

（四）乡村振兴迈出新步伐。始终按照产业兴旺、生态宜居、乡风文明、治理有效、生活富裕总要求，把乡村振兴作为高质量发展的重大战略来推进。现代农业量效齐增。全市保有耕地513.55万亩，粮食产量185.6万吨，农林牧渔业增加值195.7亿元，增长4.5%。一二三产业融合发展不断加快，省级以上现代农业产业园达73个，靖州县成功创建全省首个国家现代农业产业园，中方县获批全国农村一二三产业融合发展先导区，鹤城区获批省农村一二三产业融合发展示范县。积极推进农业品牌建设，新增“三品一标”农产品55个。“千年贡茶·怀化碣滩”推介活动在人民大会堂成功举办，靖州杨梅获评全省十大农业区域公用品牌。经营主体加速壮大。新增市级以上农业产业化龙头企业25家，农产品加工企业达3027家，实现农产品加工销售收入439亿元，增长16%。农民专业合作社增至6872家，其中国家、省级示范社分别为54家、108家。新增星级农庄18家，休闲农业实体达375家。美丽乡村亮点纷呈。创建美丽乡村示范村500余个，其中省级示范村15个。356个行政村完成农村人居环境综合整治。新建“秀美村庄”210个。113个村落入选中国传统村落名录，数量居全省首位。改造农村危房2.05万户。巩固提升农村安全饮水24.02万人，在全省率先实现全覆盖。

（五）城市建设呈现新面貌。立足提升城市形象和品位，改善宜居宜业环境，全面启动“三城同创”，成功创建省级园林城市，顺利通过省级文明城市复检。基础设施持续完善。实施城建重点项目31个，铁路编组站迁建、高堰西路等项目进展顺利。鸭嘴岩大桥实现合龙，四方路一期建成投用。应民北路、顺天南路南延段、迎丰公园北路等“断头路”即将通车。新建和改造城区大中型停车场3个，新增停车位1000余个。大湘西天然气长输管道支线项目有序推进。天网工程二期、智慧交通、智慧城管等一批智慧怀化项目建成使用。市容市貌不断改观。全力开展“绿城攻坚”行动，舞阳公园顺利竣工，新建街头小游园5个，绿化提质城市主干道3条，城区新增绿地面积近200万平米。持续加强道路、路灯等市政设施维护，怀西路、怀东路、舞水路亮化美化工程全面竣工。新建高标准公共厕所8座、旅游厕所61座。主城区拆除违法建筑6.38万平米。房地产历史遗留问题处理取得突破，盘活“烂尾楼”项目12个。城镇建设加快推进。积极推进县城提质改造，溆浦成功创建国家卫生县城，全市国家卫生县城达7个。启动第二批新型城镇化试点，全市城镇化率达48%、增长1.85个百分点。沅陵齐眉界获批国家森林小镇，芷江镇入选湖湘风情文化旅游小镇，洪江市安江镇入列省经济发达镇行政管理体制改革试点镇。

（六）民生改善交出新答卷。始终把保障和改善民生作为一切工作的出发点和落脚点，全面完成省市20项重点民生实事，财政民生支出占比达77.9%。芷江师范升格为湖湘师范高等专科学校，湖南医药学院整体搬迁进展顺利。新建城区学校9所，新增城区义务教育学位4.55万个，消除大班额2052个，其中968个超大班额全部消除到位。首次发布《怀化市就业白皮书》，新增城镇就业3.5万人、农村劳动力转移就业3.84万人，城镇登记失业率3.77%，为近年来最低。各项社会保险参保累计达941万人次，社会保险覆盖率98.5%。全市三级医院达4家，新建标准化卫生室208个，孕产妇死亡率创历史新低，活产出生缺陷发生率明显下降，计生工作保类进位。完成棚户区改造1.67万套。建成村级综合文化服务中心2727个，广播“村村响”实现全覆盖。《花瑶挑花》《侗锦织造技艺》入选首批国家传统工艺振兴目录，靖州县获评湖南民间文化艺术之乡。麻阳30周年县庆等活动成功举办。怀化代表团成年组、青少年组获省第十三届运动会金牌数第3、第6名，创历史最好成绩。居民消费价格涨幅2.2%。强力开展扫黑除恶专项斗争，社会治安综合治理工作再获全省先进。全市生产安全事故发生起数、死亡人数分别下降58.2%、57.6%。有效防控非洲猪瘟，全年未发生食品药品安全事故。同时，审计、统计、消防、人防、老龄、双拥、宗教、档案、移民、水文、气象、保险、地方志、工会、共青团、妇女儿童、外事侨务、防震减灾、优抚安置、住房公积金等各项事业和工作都取得新成效。

（七）政务服务展示新形象。坚定不移贯彻党中央国务院大政方针，不折不扣落实省委省政府和市委决策部署。建立重大事项向人大报告制度，认真落实人大及其常委会决定决议，依法接受人大法律监督和工作监督，主动接受政协民主监督。全年办理人大代表建议280件、政协委员提案262件，办结率均达100%。深化“放管服”改革，“互联网+政务服务”“四办”“多证合一”“最多跑一次”改革全面铺开。大力推行“双随机、一公开”监管工作，执法不公、执法不严问题得到有效解决。全面落实党风廉政建设“两个责任”，坚定不移纠“四风”，从严查处侵害群众利益的问题。深入开展“宣战庸懒散、提振精气神”和“马上办、钉钉子”专项行动，干部作风日趋好转。

各位代表，过去的一年，发展成果令人鼓舞，工作成绩来之不易。这些成绩的取得，最重要、最根本的是有习近平新时代中国特色社会主义思想的科学指引，也是省委省政府和市委坚强领导的结果，是市人大市政协监督支持的结果，是全市上下团结奋斗的结果。在此，我代表市人民政府，向全市广大干部群众，向各位代表、各位委员，向各民主党派、工商联、无党派人士和人民团体，向政法干警、驻怀部队官兵、消防救援指战员，向离退休老同志，向所有关心支持怀化发展的各界人士，表示衷心感谢，并致以崇高敬意！

在肯定成绩的同时，我们也清醒地认识到工作中仍存在一些问题与不足。一是经济形势稳中有变，经济下行压力加大，经济增速明显放缓，一些主要经济指标没有达到年初预期，比如固定资产投资、规模工业增加值增幅分别差3个、0.2个百分点。二是发展新动能仍然不足，产业结构不尽合理，产业发展整体水平仍然不高。三是政府债务风险、金融风险一定程度仍然存在。四是发展资金短缺，投资增速下滑趋势明显，民营经济规模不大、活力不足等问题还比较突出。五是营商环境有待优化，部分干部能力、作风和精神状态还不适应新时代新要求，不敬畏、不在乎、喊口号、装样子等形式主义、官僚主义问题依然存在，等等。对此，我们一定高度重视，认真加以解决。

二、2019年工作安排

2019年，是新中国成立70周年，是全面建成小康社会关键之年。做好全年工作，意义十分重大。

今年工作的总体要求是：以习近平新时代中国特色社会主义思想为指导，认真贯彻习近平总书记关于湖南工作的重要指示精神，坚持稳中求进工作总基调，坚持新发展理念，坚持推进高质量发展，坚持以供给侧结构性改革为主线，坚持深化市场化改革、扩大高水平开放，坚持创新引领开放崛起，继续打好三大攻坚战，继续开展产业项目建设年活动，进一步稳就业、稳金融、稳外贸、稳外资、稳投资、稳预期，加快推进“一极两带”和“一个中心、四个怀化”建设，提高人民群众获得感、幸福感、安全感，保持经济持续健康发展和社会大局稳定，奋力谱写建设富饶美丽幸福新湖南的怀化篇章，为全面建成小康社会收官打下决定性基础，以优异成绩庆祝中华人民共和国成立70周年。

经济社会发展主要预期目标是：地区生产总值增长8%，规模工业增加值增长7.5%，固定资产投资增长12%，社会消费品零售总额增长10.5%，地方公共财政收入增长6%，进出口总额增长20%，城乡居民人均可支配收入分别增长8.5%、10.5%，居民消费价格涨幅控制在3.0%左右，万元GDP能耗下降3.2%。

实现上述目标，必须重点抓好七个方面工作。

（一）继续打好三大攻坚战。按照已确定的行动方案，针对突出问题，打好重点战役。

1．坚决打好防范化解重大风险攻坚战。完善全市政府隐性债务化解方案，积极推动资源资产化、资产资本化、资本证券化，有序有效偿还隐性债务，确保隐性债务总量不增长、借新还息不发生、“三保”资金不断链。依法依规实施政府购买服务、政府投资基金项目。严格执行“停缓调撤”要求，科学确定融资规模。加快平台公司实质性转型，实现政企分开。健全应急处置预案，加强互联网金融领域、民间融资市场监管，依法严厉打击非法集资。坚决打击恶意逃废债行为，努力降低不良贷款率，营造良好金融生态。

2．坚决打好精准脱贫攻坚战。实现7万人以上农村贫困人口脱贫，84个贫困村出列，沅陵、溆浦、麻阳、通道4县脱贫摘帽，贫困发生率降至0.6%左右。完善“四跟四走”产业扶贫机制，全面开展贫困村产业空白村清零行动。推进就业扶贫，新建一批“扶贫车间”，稳定扶贫特岗1万个。做好易地扶贫搬迁后续工作，全面完成农村危房改造任务。不折不扣落实生态扶贫、教育扶贫、健康扶贫政策。完成25户100人以上自然村通水泥路1000公里、农村公路提质改造500公里。实施行政村农网改造141个，自然村光纤覆盖率达55%。继续推进农村安全饮水巩固提升工程。全面巩固脱贫成果，强化动态管理，抓好返贫人口脱贫工作。建立稳定脱贫长效机制，扎实开展“回头看、回头查、回头帮”，减少和防止贫困人口返贫。注重扶贫同扶志、扶智相结合，激发贫困群众内生动力。

3．坚决打好污染防治攻坚战。扎实推进蓝天保卫战，深入开展扬尘综合治理，落实裸露泥土全覆盖、土方开挖湿法作业、渣土运输整治以及大气污染联防联控等措施，确保PM10明显改善，城区空气质量优良率稳定在92%以上。扎实推进碧水保卫战，全面落实河长制，深化太平溪综合治理，确保13条黑臭水体整治达标，积极创建国家水生态文明示范城市；加强饮用水水源保护，确保全市地表水考核断面水质达标率95%以上，集中式饮用水水源保护区水质达标率保持100%。扎实推进净土保卫战，加强重点区域土壤污染治理与修复，有效管控农用地和城市建设用地土壤环境风险，坚决完成重金属污染专项治理任务。统筹推进山水林田湖草系统生态保护与修复，继续实施1000公里高速高铁绿化提质工程、1000个秀美村庄建设工程、1000万亩封山育林工程，完成人工造林25万亩。

（二）扎实推进产业及产业园区三年提升行动。继续开展产业项目建设年活动，实施重点产业项目200个，年度投资300亿元。

1．推动制造业高质量发展。以国家承接产业转移示范区建设为抓手，做好建链、补链、强链文章，加快培育电子信息、新材料、新能源等产业集群。启动高新区中金润电子信息产业园、沅陵省级电子信息产业园建设，加快经开区电子信息科技园建设，培育壮大奇力新、华晨、优美兴科技、金升阳、合利来电子等企业，力争电子信息产业主营业务收入增长20%。依托久日新材、四维住工、怀化远大、宇隆电工等重点企业，做大做强新材料产业。依托高新区新能源汽车、力可泰动力电池、新能源环保型产业基地等重点项目，加快发展新能源产业。

2．推动第三产业快速壮大。以商贸服务型国家物流枢纽承载城市建设为抓手，引导现代物流企业向经开区和重点园区集聚，提升狮子岩物流园智能化、国际化水平，抓好高新区工业物流园、经开区综合口岸枢纽工程、传化智能公路物流港、佳惠·武陵山农产品（冷链）物流产业园、京东怀化数字经济产业园等项目，加快建设西南一流商贸物流基地。推动通道侗文化旅游区、洪江古商城、芷江抗战胜利受降纪念地、黔阳古城创建5A级景区，沅陵借母溪创建4A级景区，启动共建雪峰山国家山地旅游度假区，加快具备五星级条件酒店建设，力争实现接待游客6500万人次、旅游收入510亿元，积极创建国家全域旅游示范市。推进高新区医药健康产业园、经开区综合健康养老服务中心、通道北大未名生物科技园等项目建设，支持正清、正好、怀仁、天骑、补天、林泉药业等企业发展，全力争创全国医药健康产业示范基地。

3．推动园区专业化建设。充分发挥高新区、国家农业科技园区、经开区引领作用，按照“一县一产业”“一园区一特色”思路，围绕专业化，打造全产业链，推动沅陵电子信息、辰溪军民融合、靖州绿色食品等专业化园区发展，引导13个工业集中区错位发展。落实省“135”工程升级版要求，新增标准化厂房100万平米、入园项目100个，实现园区技工贸收入增长18%，园区规模工业增加值增长8.5%。鼓励园区建设多层标准厂房，提高园区亩均产出、亩均税收。力争高新区、经开区和部分县级工业集中区跻身全省改革创新试点园区。

（三）全面落实创新引领开放崛起战略。推动生态创新融合发展，深化市场化改革，扩大高水平开放，加快建成国家创新型城市和湖南西南开放门户。

1．深化供给侧结构性改革。遵循“巩固、增强、提升、畅通”方针，完成“三去一降一补”任务。巩固煤矿、小煤窑、非煤矿山等领域治理成果，坚决防止已退出落后产能死灰复燃。加大“僵尸企业”出清力度，稳妥分流安置职工。构建房地产市场健康发展长效机制，不断完善住房市场体系和保障体系。持续降低企业生产运营成本，力争低于沿海地区5%-10%。深入实施质量强市战略，新增一批国家、省级知名品牌。深化市管企业改革，推动从管企业向管资本转变。全面完成政府机构改革。推进农村土地“三权分置”，全面完成农村承包地确权登记颁证。稳步实施全国集体林业综合改革试验区、“两权”抵押贷款增量扩面、乡镇供销社恢复、公共资源管理、城管体制、多县一行制村镇银行试点、分级诊疗制度等重点改革。

2．加快生态创新融合发展。加大领军企业培育力度，新增高新技术企业75家。突出企业研发投入主体地位，力争R&D经费支出33亿元。发挥中国西南高新技术成果转化孵化基地作用，加快建设怀化科技港。引导辰溪、靖州、新晃等工业集中区申报省级高新区。培育国家级知识产权优势企业3家以上，力争每万人发明专利拥有量达1件以上，园区企业专利产品销售额占总销售额50%以上。引进和培养科技创新人才20名以上。

3．着力打造内陆开放新高地。深入开展“五大开放行动”，力争引进世界500强、中国500强、中国民营500强企业10家以上，招商引资省外境内资金320亿元，增长18%以上。加强企业上市辅导工作，全力支持企业上市。强化铁路口岸、舞水公共保税仓、跨境商品展示交易中心等平台支撑作用。积极筹划怀商大会，鼓励引导广大怀商回故乡建家乡。着力稳外贸，力争全市进出口总额突破1亿美元。

（四）深入推进乡村振兴战略。落实乡村振兴战略规划，细化实化工作举措，不断巩固发展“三农”持续向好形势。

1．着力推进农业现代化。立足产业兴旺，严守382.86万亩永久基本农田红线，种植优质稻190万亩。加快发展“一县一特”，做大做强茶叶、新晃黄牛、芷江鸭等10大区域特色产业。推动全域中药材种植，完成种植面积48万亩，综合产值90亿元。培育新型经营主体，新增龙头企业15家、农产品加工企业100家以上，实现加工销售收入500亿元以上；新增家庭农场200个、农民专业合作社300个以上，争创省级以上农民专业合作示范社5个。加快鹤城九丰、新晃绿丰、沅陵辰龙关等特色产业园建设，推进芷江两岸精致农业示范园建设，创建省级特色产业园5个。积极申报麻阳黄桃、溆浦瑶茶、会同竹笋、芷江甜茶等国家地理标志保护产品，新增“三品一标”30个。

2．着力推进美丽乡村建设。着眼生态宜居，推行“多规合一”，加快编制全域国土空间规划。打好农村人居环境整治首仗，推进农村“厕所革命”，实现农户卫生厕所普及率50%以上；完成行政村生活垃圾治理341个、生活污水治理181个、农村环境综合整治234个，创建省级美丽乡村示范村15个。推进公路安全生命防护工程，全面铺开城乡客运一体化工作。认真开展全市第三次国土调查，全面清理全市闲置土地，做好城乡建设用地增减挂钩和耕地占补平衡工作。

3．着力推进农村移风易俗。加快制定完善村规民约，深化文明村镇、文明家庭、文明集市创建。广泛开展好媳妇、好儿女、好公婆等评选表彰活动，倡导勤劳致富、尊老爱幼、厚养薄葬等社会风尚。抓好乡镇文化站、农村综合文化服务中心建设，丰富乡村文化生活，提高村民文明素养，提升乡风文明程度。

（五）持续开展“三城同创”。全力创建全国文明城市、国家卫生城市、国家园林城市和国家森林城市，力争年内获得国家卫生城市申报资格，不断提升城市环境品质和市民生活质量。

1．完善城市基础设施。抓好《怀化市城市总体规划（2019-2035）》编制。严格查违拆违，铁腕整治违建行为。继续推进锦绣五溪、恒大、万达商业广场、步步高等新型城市综合体和新兴商圈建设。加快推进主城区应急水源建设，抓紧实施城区供水管网工程。启动生活垃圾焚烧发电厂建设，新建和改扩建一批城市公厕和垃圾中转站。完成锦园路、吉祥大道、顺达路等城区道路建设，加快正清路、红星南路等道路提质改造。抓好棚户区改造、公租房建设及后续管理。新建停车场4个，新增停车位2500个。

2．提升城市功能品质。扎实推进“绿城攻坚”行动，实施中坡森林公园、城郊国省干道绿化提质改造，完成岩门公园、太平溪风光带、舞水河风光带建设，加快迎丰公园二期建设，新增城区绿地面积327万平米。实施天网工程三期、“互联网+政务服务”、大数据双创中心等“智慧怀化”项目。推进城区农贸市场提质改造。强化“门前三包”，综合整治市容市貌、交通秩序、环境卫生等，提高城市精细化管理水平。

3．加快城镇体系建设。引导支持县城提质扩容，高品质建设新城区。加快建设沅陵官庄、辰溪孝坪、溆浦低庄、麻阳锦和、鹤城黄金坳、通道县溪等一批重点特色镇。抓好已在城镇就业的农村转移人口的落户工作，全市城镇化率提高到49.5%。

（六）进一步稳投资促消费。发挥投资关键作用，扩大居民消费需求，激发民营经济活力，不断增强区域发展内生动力。

1．稳定投资需求。突出项目带动，重点推进重大项目300个，年度投资550亿元。巩固全国性综合交通枢纽城市地位，加快怀化西铁路编组站迁建、张吉怀客运专线、渝怀铁路增建二线、焦柳铁路怀化段电气化改造、怀化动车所建设，启动高铁南站改扩建、怀化东货场建设；力争沅辰、靖黎、芷铜高速开工建设，推动张吉怀高铁南延线、溆洞高速前期工作；实施沅水辰溪至洪江段航道整治工程；完成芷江机场航站楼改扩建主体工程，实现旅客年吞吐量60万人次。力争建成运行大湘西天然气长输管道支线项目。新建抗旱应急水源工程8500处，实施水毁工程修复7210处，完成病险水库除险加固18座。

2．增强消费拉动力。升级汽车、餐饮、居住等实物消费，拓展家政、健康、养老等新型消费，加快教育、育幼、医疗、文化等服务业发展。推动实体商业与互联网有效融合，实现电子商务交易额增长35%。办好生态文化旅游节、汽车博览会、健康产业博览会、农博会等节会活动，促进会展经济快速发展。落实个人所得税专项附加扣除政策，增强消费能力。

3．支持民营经济发展。落实国家减税降费政策，清理规范收费项目，切实减轻企业负担。加强财政、信贷、产业政策联动，组织银企对接会，打通实体经济融资“最后一公里”。坚决防止政府部门和国有企业违约拖欠中小企业款项。依法依规保护民营企业知识产权和民营企业家合法权益。积极支持本土企业拓展本地市场。建立健全民营企业联系制度，构建亲清新型政商关系。

（七）加强保障和改善民生。坚持以人民为中心，精心办好一批重点民生实事，不断实现人民群众对美好生活的向往。

1．把稳就业摆在突出位置。抓好高校毕业生、农民工、退役军人、贫困劳动力等重点群体就业创业，确保零就业家庭动态清零。完善“互联网+就业服务”平台，引导和支持各县市区产业园区建设创业孵化基地。新增城镇就业3.5万人、农村劳动力转移就业3.8万人，城镇登记失业率控制在4.5%以内。

2．努力办好人民满意的教育。深入实施消除大班额工作专项行动，新增义务教育学位3.14万个，消除大班额1292个。加大配建学校建设力度，积极引导名校来怀投资办学。推动中方等县6所芙蓉学校投入使用，会同等县4所芙蓉学校全部开工。加强乡村小规模学校（幼儿园）建设，新建标准化村小教学点185所、农村公办幼儿园20所。推进怀化职教资源整合，加快市级职教公共实训基地建设。积极推进湖湘师范高等专科学校软硬件升级。稳定乡村教师队伍，加强师德师风建设。

3．加快发展卫生、文化等社会事业。加强全科、儿科、妇产科等紧缺专业能力建设，提高县级医院综合能力，加快乡镇卫生院规范化建设，建成标准化卫生室29个。加强疾病防控和卫生应急。持续降低出生人口性别比和出生缺陷发生率。推动县级图书馆、文化馆达标升级建设。支持通道侗族村寨申报世界文化遗产。广泛开展群众文化活动、全民健身活动。加强高水平体育后备人才培养，提升竞技体育水平。

4．加强和创新社会治理。扎实推进扫黑除恶专项斗争和“集打斗争”，深入开展“扫黄打非”，积极创建全国禁毒示范城市。聚焦安全生产“三下降一杜绝”目标，坚决遏制较大以上安全事故发生。强化食品药品安全监管工作，确保人民群众饮食用药安全。建立健全应急管理体系，抓好防灾救灾和应急处突能力建设。及时回应群众关切，扎实做好信访维稳工作，不断增强人民群众的安全感。

同时，统筹落实社保惠民新政，扩大社会保障受益面；重视城乡困难群众帮扶解困，完善临时救助工作制度；协调推进国防动员、民兵预备役、人民防空、退役军人事务等工作，推动军民融合发展；认真开展第四次全国经济普查；支持工会、共青团、妇联等群团组织工作；做好民族宗教、外事侨务、老龄老干、审计、残联、档案、保密、气象、保险、水文、移民、防震、地方志、住房公积金等工作。

三、进一步加强政府自身建设

新的一年，全市政府系统将进一步弘扬实干精神，强化责任担当，以钉钉子精神推动各项工作高效落实，切实做到忠诚、干净、担当。

（一）提升站位抓落实。务必把政治建设摆在首位，旗帜鲜明讲政治。要坚定不移树牢“四个意识”，坚定“四个自信”，坚决做到“两个维护”，自觉用习近平新时代中国特色社会主义思想武装头脑。坚定不移贯彻省委省政府和市委决策部署，不折不扣落实创新引领开放崛起战略，推动高质量发展。真正做到信念过硬、政治过硬、责任过硬、能力过硬、作风过硬，以实际行动落实党对经济工作的领导。

（二）依法行政抓落实。务必进一步强化法治思维，切实把政府各项工作纳入法治轨道，建设法治政府。要落实重大决策事项向人大报告制度，依法接受人大及其常委会法律监督和工作监督，认真办理人大代表建议和人大常委会审议意见；主动接受政协民主监督，虚心听取和采纳政协委员、民主党派、工商联、无党派人士和人民团体的意见建议；深入推进“七五”普法，严格规范公正文明执法。真正做到法无授权不可为，法定职责必须为，想问题、作决策、办事情自觉带头守法律、重程序、受监督。

（三）主动作为抓落实。务必树牢不进则退的危机意识，以时不我待、只争朝夕的精神投入工作。要进一步强化等不起、慢不得的紧迫感，坚决克服慢半拍、混日子现象；进一步强化出新招、求实效的使命感，坚决破除思维定势、工作惯性和路径依赖，不断开拓创新，建设创新政府；进一步强化勇任事、敢担当的责任感，坚决整治敷衍塞责、推诿扯皮的不良风气。真正做到雷厉风行不拖延，合力攻坚不推诿，坚决破除形式主义、官僚主义。

（四）优化服务抓落实。务必强化服务意识，增强服务能力，建设高效政府和服务型政府。要强化市场监管，坚决清理废除妨碍统一市场和公平竞争的各项规定和做法，营造公开公平公正的投资环境；加强学习培训和调查研究，增强适应新形势新任务的能力和水平；实行行政审批中介服务清单制度，压缩审批时限，提高各类行政审批效率；加强诚信政府建设，重点整治危害群众利益、损害市场公平交易等失信行为。真正做到该办的事项，件件快办；确定的项目，个个见效；作出的承诺，项项兑现。

（五）严守底线抓落实。务必将“严”字长期坚持下去，持之以恒正风肃纪，建设廉洁政府。要坚决落实中央八项规定和省委实施办法，严格执行廉洁从政各项规定，落实党风廉政建设主体责任和“一岗双责”；盯紧看牢政府采购、工程招投标、土地出让等重点领域和关键环节，从源头堵住廉政风险漏洞；严厉惩治各类腐败行为，坚决整治“雁过拔毛”等发生在群众身边的不正之风。真正做到明大德、守公德、严私德，以为民务实清廉形象赢得人民群众信任和支持。

各位代表！

今天的怀化，前进的步伐铿锵有力，发展的潜力前所未有。让我们紧密团结在以习近平同志为核心的党中央周围，在省委省政府和市委的坚强领导下，坚定信心，奋发图强，加快推进“一极两带”和“一个中心、四个怀化”建设，以优异成绩迎接中华人民共和国成立70周年！
